# RNAi-Mediated c-Rel Silencing Leads to Apoptosis of B Cell Tumor Cells and Suppresses Antigenic Immune Response In Vivo

**DOI:** 10.1371/journal.pone.0005028

**Published:** 2009-04-06

**Authors:** Wenzhi Tian, Hsiou-Chi Liou

**Affiliations:** Division of Immunology, Department of Medicine, Weill Medical College of Cornell University, New York, New York, United States of America; Ordway Research Institute, United States of America

## Abstract

c-Rel is a member of the Rel/NF-κB transcription factor family and is predominantly expressed in lymphoid and myeloid cells, playing a critical role in lymphocyte proliferation and survival. Persistent activation of the c-Rel signal transduction pathway is associated with allergies, inflammation, autoimmune diseases, and a variety of human malignancies. To explore the potential of targeting c-Rel as a therapeutic agent for these disorders, we designed a small interfering RNA (siRNA) to silence c-Rel expression in vitro and in vivo. C-Rel-siRNA expression via a retroviral vector in a B cell tumor cell line leads to growth arrest and apoptosis of the tumor cells. Silencing c-Rel in primary B cells in vitro compromises their proliferative and survival response to CD40 activation signals, similar to the impaired response of c-Rel knockout B cells. Most important, in vivo silencing of c-Rel results in significant impairment in T cell-mediated immune responses to antigenic stimulation. Our study thus validates the efficacy of c-Rel-siRNA, and suggests the development of siRNA-based therapy, as well as small molecular inhibitors for the treatment of B cell tumors as well as autoimmune diseases.

## Introduction

RNA interference (RNAi)-mediated gene silencing has been a powerful approach for functional studies of a particular gene in biological systems [1], [2]. At present, several clinical trials are ongoing to test small interfering RNA (siRNA)-based therapies for age-related macular degeneration and viral diseases [1], [2]. The Rel/NF-kB transcription factors have been considered important therapeutic targets, because their persistent activation can lead to tumorigenesis, inflammation, and autoimmunity [3]–[8]. The purpose of this report is to provide a proof-of-concept study to demonstrate the feasibility of targeting the c-Rel member of the Rel/NF-κB family as potential therapy for B cell tumor and inflammatory disorders.

The mammalian Rel/NF-kB transcription factor family contains five members: c-Rel, p50, p65, RelB, and p52. Due to their differential tissue expression pattern and target gene specificities, the five Rel/NF-kB members play distinctly unique roles in biology and disease [8]–[12]. Earlier studies have shown that p50 and p65 are ubiquitously expressed in all tissue types, whereas the other three members (c-Rel, p52, and RelB) are predominantly expressed in differentiated lymphoid and myeloid cells [13]–[16]. Subsequent studies using gene targeting approaches further demonstrated the distinctive phenotype and disease susceptibility of individual Rel/NF-kB knockout mice. For example, due to restricted expression of c-Rel, RelB, and p52 in hematopoietic cells, these knockout mice are viable and only exhibit impairment in the immune cells [17]–[25]. By contrast, p65 knockout mouse exhibit early embryonic lethality resulting from extensive hepatocyte apoptosis [26], [27].

Substantial evidence has further suggested that c-Rel is particularly noteworthy as a desirable therapeutic target, among the Rel/NF-kB family. The c-Rel proto-oncogene is the cellular counterpart of the v-Rel oncogene originally discovered in an avian retrovirus that causes acute lymphoma in chickens [7], [28]. C-Rel gene amplification or persistent activation has been detected in many human B cell tumors, including diffuse large B cell lymphomas, primary mediastinal lymphoma, CLL, and multiple myeloma, as well as in some solid tumors [29]–[45]. Perhaps the most intriguing findings come from a series of studies assessing the tumorigenic potential of the Rel members. The systematic analyses unequivocally demonstrate that c-Rel is the most oncogenic member among the Rel/NF-kB family [28], [29], [31], [32], [46], [47], thus supporting its critical role in tumorigenesis.

The role of c-Rel in biology and disease has also been addressed by the use of c-Rel knockout mice. Since c-Rel expression is restricted to mature hematopoietic cells, c-Rel knockout exhibit deficiencies only in the immune response to antigens [8], [48]–[51]. Otherwise, the c-RelKO mice are viable and have a normal life span. Due to c-Rel defects in lymphocytes and myeloid cells, the c-RelKO mice do not develop allergic inflammation, autoimmune diseases (EAE, Type I diabetes, collagen induced arthritis), or reject allogeneic transplants [17]–[22]. Nonetheless, their innate immune responses to pathogens (e.g. influenza virus, Toxoplasma gondii, Listeria monocytogenes) remain largely intact [52]–[54], suggesting that blocking c-Rel pharmacologically may not cause severe global immunosuppression.

c-Rel is involved in autoimmune diseases and cancer via regulation of the expression of cytokines, anti-apoptotic molecules, and cell cycle regulators. At least 11 cytokine genes have been shown to be c-Rel targets, including TNF-α, IL-1, IL-2, IL-6, IL-10, IL-12, IL-15, IL-17, IL-23, IL-27, and IFN-γ. In addition, c-Rel controls the expression of cell cycle molecules (E2F3a, cyclin D2/3, cyclin E), survival proteins (BclX, Bfl1, Mcl-1), signaling molecules, growth factors, and transcription factors [18], [19], [48], [50], [55]–[68]. Therefore, the c-RelKO mouse as well as oncogenic transformation studies support the rationale for considering c-Rel as an attractive therapeutic target for autoimmune/inflammatory diseases and B cell tumors, in that blocking c-Rel (i) may inhibit the production of inflammatory cytokines and tumorigenic factors, but (ii) does not cause systemic tissue toxicity, as c-Rel function is confined to the hematopoietic cells, and (iii) would not cause global immunosuppression, since innate immunity against pathogens remains largely intact in c-RelKO mice.

To explore the therapeutic potential of targeting c-Rel, we designed a small interfering RNA (siRNA) to silence c-Rel in B cell lymphoma as well as in immune cells. Our data reveal that, upon silencing c-Rel in a B cell tumor line, the cells undergo growth arrest and apoptosis in a dose-dependent manner. Intriguingly, in vivo silencing c-Rel renders the mice less responsive to antigenic stimulation, mimicking the impaired immune phenotype of the c-Rel knockout mice.

## Results

### Generation of c-Rel silencing retroviral construct

One of the hurdles in developing siRNA-based therapy is delivery of siRNA into cells and tissues. Since our initial purpose was to validate c-Rel as a potential therapeutic target, we utilized a retrovirus to deliver siRNA, as these agents have a higher transduction efficiency in tumor cell lines and primary cells, which are often resistant to conventional DNA transfection methods.

**Figure 1:**
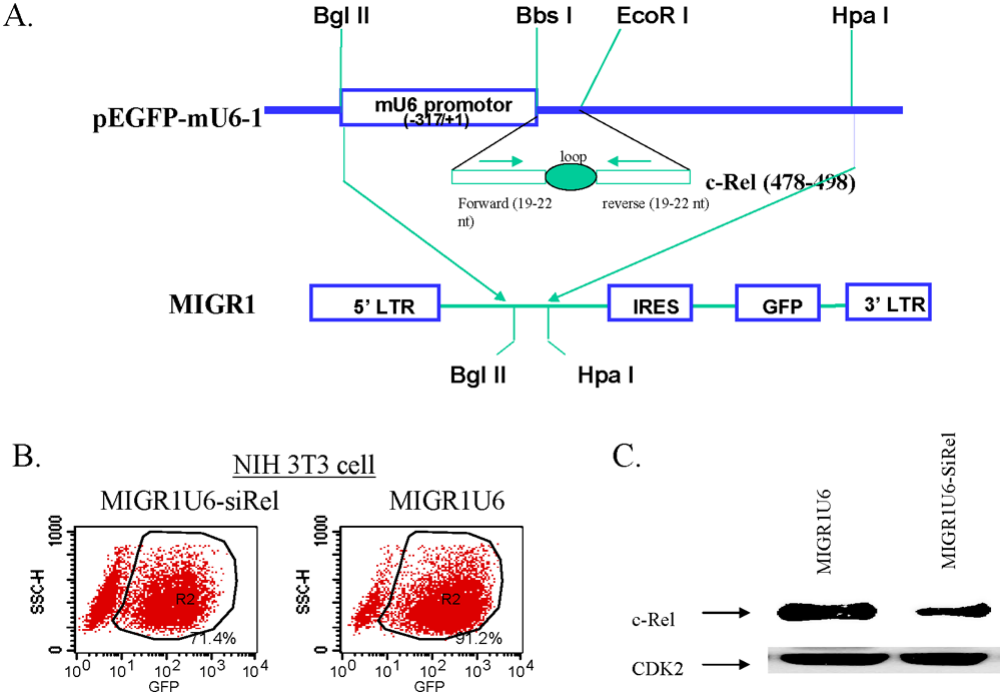
In vitro silencing of c-Rel. (a) Diagram of retroviral vector and c-Rel-siRNA. (b) 2×10^5^ of NIH3T3 cells in 4 ml of DMEM were seeded in 6-cm dishes and were cultured for 24 hours before addition of 100 µl of c-Rel siRNA expressing retrovirus or control virus (virus titer: 5×10^6^/ml). At 48 hours post-infection, cells were harvested and monitored by flow cytometry for the infection efficiency. (c) Cell lysates were prepared from the virus-infected NIH3T3 cells. 30 µg of the cell lysates were loaded onto 12% SDS-PAGE gel that was transferred onto nylon membrane. The membrane was Western blotted with c-Rel specific polyclonal antibody. After stripping off the c-Rel specific antibody, membrane was re-blotted with CDK2 specific antibody. When normalized to CDK2 level, the c-Rel expression in the MIGR1U6 and MIGR1U6-siRel group is 1.22 and 0.43, respectively, indicating ∼65% reduction of c-Rel expression in this particular c-Rel silenced sample.

To generate a c-Rel-siRNA, we selected a 21-nucleotide (nt) sequence unique to murine c-Rel coding sequences that would enable efficient knockdown of its mRNA using the rules solicited by previous empirical studies [69]–[72]. The c-Rel-silencing oligonucleotides were first cloned into the pEGFP-mU6-1 vector, immediately downstream of its U6 promoter (**Fig. 1a**) [73], [74]. The siRNA-expressing cassette was then sub-cloned into the *Bgl* II and *Hpa* I sites of the MIGR1 vector to generate a new construct, MIGR1-mU6-siRel. The presence of an IRES sequence followed by the green fluorescent protein (GFP) gene in the MIGR1 vector allows a co-expression of c-Rel siRNA duplex and the GFP, the latter being used for monitoring transduction efficiency. This vector was co-transfected with two packaging plasmids into 293T cells, following the procedure described in our previous studies [73]–[75]. Virus was harvested from the cell culture supernatant and contained a titer of ∼5×10^6^/ml.

### Silencing of c-Rel

To test the silencing effect of the c-Rel-siRNA retrovirus, we utilized NIH3T3 cells that express c-Rel constitutively. The cells were cultured for 24 hours prior to virus infection. Two days post-infection, cells were harvested and analyzed for percentage of GFP+ cells by flow cytometry to quantify infection efficiency (**Fig. 1b**). The infection efficiencies for c-Rel-siRNA and control viruses were 71% and 91% respectively. Cell lysates were then prepared and subjected to Western blot analysis for c-Rel protein expression. By densitometry, a 70% reduction of c-Rel protein was observed in the cells transduced with c-Rel-siRNA as compared to the control cells (**Fig. 1c**), indicating that the siRNA is effective in reducing c-Rel expression.

### Silencing c-Rel resulted in diminished cell survival and cell cycle progression of a B cell tumor line

**Figure 2:**
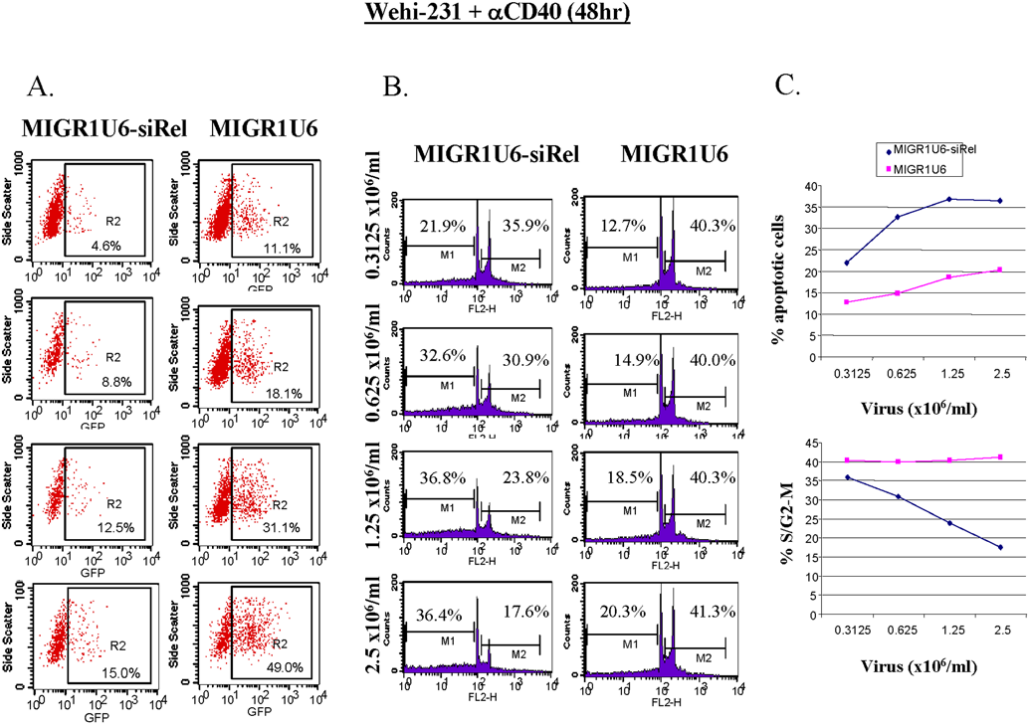
Silencing c-Rel resulted in diminished cell survival and cell cycle progression in Wehi-231 cells. (a) 2 ml of Wehi-231 cells (2×10^6^/ml) were cultured in a 6-well plate with anti-CD40 (10 µg/ml) for 48 hours in the presence of polybrene (4 µg/ml) and different dosages of the c-Rel siRNA expressing retrovirus or the control virus (0.3125, 0.625, 1.25, and 2.5×10^6^). Cells were harvested and monitored by flow cytometry for the percentage of GFP^+^ cells. c-Rel silencing was confirmed by western blot analysis. (b) Cell survival and cell cycle progression was analyzed by PI staining. The number of percentage in M1 indicates the percentage of the analyzed cells undergoing apoptosis. The number in M2 representing the number of cells entering into cell cycle. (c) Data in (b) were summarized as line chart type figure.

c-Rel overexpression or gene amplification has been reported in a variety of B cell tumors [29]–[32]. To validate c-Rel as a potential drug target for B cell tumors, we applied c-Rel-siRNA to B cell tumors expressing constitutive c-Rel activity. The murine B cell tumor line, Wehi-231, expresses high levels of c-Rel and p50 that are required for maintaining its survival and proliferation [13], [76]–[86]. To facilitate retroviral infection efficiency and integration into cycling cells, we utilized anti-CD40 to stimulate the proliferation of Wehi-231. Another reason we chose anti-CD40 is that CD40 signaling pathway leads to the activation of c-Rel, thus allowing us to investigate the role of c-Rel in B cell proliferation. We observed a viral dose-dependent increase in infection efficiency for both samples transduced with either cRel-siRNA or control viruses, as measured by GFP+ cell percentage (**Figure 2a**). Although the infection efficiency was comparable for both viral stocks, there was a noticeably lower percentage of GFP+ population in the c-Rel-siRNA infected Wehi-231 sample compared to the controls (e.g. 15% vs 49% at 2.5×10^6^/ml sample) forty eight hours post-infection. Since c-Rel is required for the expression of Bcl-X, c-myc, and other survival molecules in Wehi-231 cells [76], [77], [79], [80], [87], [88], the lower percentage of GFP+ cells in the c-Rel-siRNA samples may be indicative of cell apoptosis induced by the expression of the specific siRNA. To further investigate this issue, we analyzed the cells by propidium iodide (PI) staining to quantify cell survival and proliferation. We observed a dose-dependent reduction in cell survival in the c-Rel silencing groups (**Fig. 2b, 2c**). Concomitant with increased apoptosis, the c-Rel silencing groups also have significantly reduced cell cycle progression, as enumerated by the percentage of cells in the S/G2/M phase. An attempt to perform PI and GFP double staining was unsuccessful due to an interference of GFP signal by PI. Nonetheless, our data confirmed the role of c-Rel in maintaining cell growth and survival of Wehi-231 tumor cell line and validated the use of c-Rel-siRNA in inducing growth arrest and apoptosis of B cell tumors with constitutive c-Rel activity.

### Silencing c-Rel in primary B cells leads to decreased cell survival and proliferative response to mitogenic stimulation

**Figure 3:**
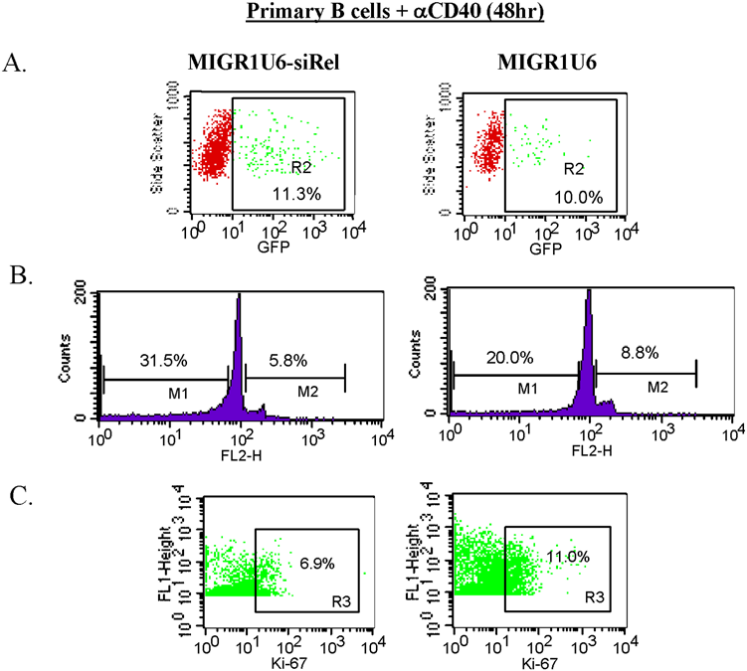
In vitro silencing of c-Rel led to impaired cell survival and cell cycle progression in primary B cells. (a) Primary B cells isolated from mouse spleen were stimulated for 24 hr with anti-CD40 (10 µg/ml) before addition of the retroviruses. Cells were harvested at 48 hr post-infection and were monitored by flow cytometry for the infection efficiency. (b) Cell survival and cell cycle progression was analyzed by PI staining. (c) Cells harvested from the same culture in (a) were stained with anti-Ki-67 using intracellular staining methods and analyzed by flow cytometry. The number represents the percentage of Ki-67 positive cells within the GFP^+^ population.

We and others have previously utilized the c-Rel knockout mice to show that blocking c-Rel in B and T lymphocytes resulted in impaired cell proliferation and survival response to mitogenic and antigenic stimulation [48], [50], [51], [55], [56], [59]–[62], [64], [85], [89]–[92]. To further validate the use of c-Rel-siRNA in primary cells, we stimulated primary B cells with anti-CD40 to trigger cell proliferation 24 hours prior to infection with either the control or c-Rel siRNA expressing retrovirus. We anticipated that silencing c-Rel in primary B cells should render the cells less responsive to mitogenic stimulation, just like c-Rel knockout lymphocytes. In both control and the c-Rel-siRNA groups, only about 11% of the cells could be infected (**Figure 3a**). The percentage is consistent with the portion of cells entering the cell cycle during a 48-hour activation period. Nonetheless, we observed that B cells infected with c-Rel siRNA virus had an increased percentage of apoptotic cells (31.5%) and decreased percentage of cells in cell cycle progression (5.8%), compared to the control group (20%, 8.8%), as measured by PI staining (**Fig. 3b**). Since PI emission wavelength overlaps with that of GFP, it was impossible to distinguish the proliferative/survival events within GFP+ cell population. Therefore, we utilized anti-Ki-67 to detect the nuclear antigen Ki-67, which is present during all active phases of the cell cycle (G1, S, G2, and mitosis), but is absent from resting cells (G0). A secondary antibody conjugated with allophycocyanin (excitation 650, emission 660) was then used to detect Ki-67-expressing cells. By gating on the GFP+ population indicative of retroviral infected cells, we observed a decreased staining of Ki-67 in the c-Rel silencing group as compared to the control group (6.9% vs 11%) (**Fig. 3c**). These results are consistent with the reduced cell growth and survival response to CD40, antigenic, and mitogenic signals in B cells derived from the c-Rel knockout mice [48], [50], [51], [55], [56], [59]–[62], [64], [85], [89]–[92].

### c-Rel knockdown lymphocytes have impaired proliferative and survival responses to mitogenic signals

To further validate the use of c-Rel-siRNA in the context of the entire animal, we generated c-Rel knockdown chimeric mice by reconstituting the bone marrow of irradiated recipient mice with the bone marrow cells infected with c-Rel-siRNA or control viruses, as previously described [73]–[75] (also see Materials and Methods). Two months post-reconstitution, some chimeric mice from a group of 8–10 mice per experiment were used for in vitro analyses and the others were used for antigen immunization experiments. Reconstitution of lymphoid cells in the chimeric mice by donor bone borrow cells were enumerated. Total cell number in spleen is 110–130×10^6^, with an average of 30–50% GFP+ cells in both T and B cell lineages (data not shown). Additionally, there is no significant difference in the total lymphoid cell number or GFP+ percentage between c-Rel-siRNA and MIGR1 transduced mice. This is consistent with previous findings that c-Rel knockout mice have normal development of B and T cell lineage [48], [50].

**Figure 4:**
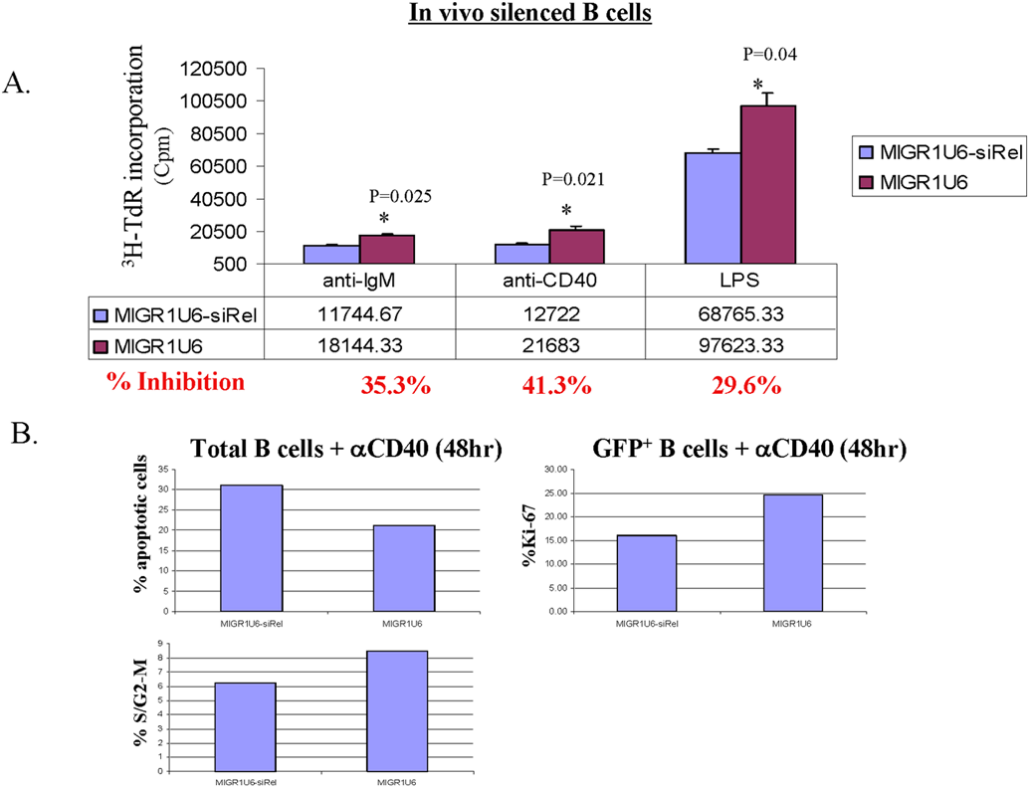
In vivo silencing c-Rel led to impaired cell survival and cell cycle progression in primary B cells. (a) Primary B cells isolated from the chimeric mouse spleen (either silencing mice or control mice) (See Materials and Methods) were stimulated for 48 hr respectively with anti-IgM (10.0 µg/ml), anti-CD40 (10.0 µg/ml), and LPS (10.0 µg/ml). Cells were pulsed with 0.5 µci of ^3^H-thymidine for 6 hours before harvest and assayed for ^3^H-thymidine incorporation. Total cell number in spleens of reconstituted mice is 110–130×10^6^, with an average of 30–50% GFP+ cells in both T and B cell lineages. The total lymphoid cell number or GFP+ percentage between c-Rel-siRNA and MIGR1 transduced mice are comparable. (b) B cells stimulated with anti-CD40 in (a) were further analyzed respectively by PI staining for cell survival and cell cycle progression, and by Ki-67 staining for cell proliferation.

Splenic B cells from the chimeric mice were stimulated for 48 hr with different mitogens: anti-IgM, anti-CD40, and LPS. Cell proliferation was monitored by thymidine incorporation. As shown in **Figure 4a**, the c-Rel silencing group had a reduced cell proliferation in response to all three mitogens tested. The percentage of inhibition was 35.3% (anti-IgM), 41.3% (anti-CD40), and 29.6% (LPS), respectively. The reduced response to CD40 signaling was further confirmed by PI staining analysis, which showed that over 10% more cells underwent apoptosis and fewer cells entered into cell cycles in the c-Rel silencing group. When only the GFP+ population was analyzed by Ki-67 staining assay, there were significantly less proliferative cells in the c-Rel-siRNA group than the control group (15.5% vs 25%) (**Fig. 4b**).

### c-Rel-siRNA chimeric mice exhibited impaired immune responses to antigen

**Figure 5:**
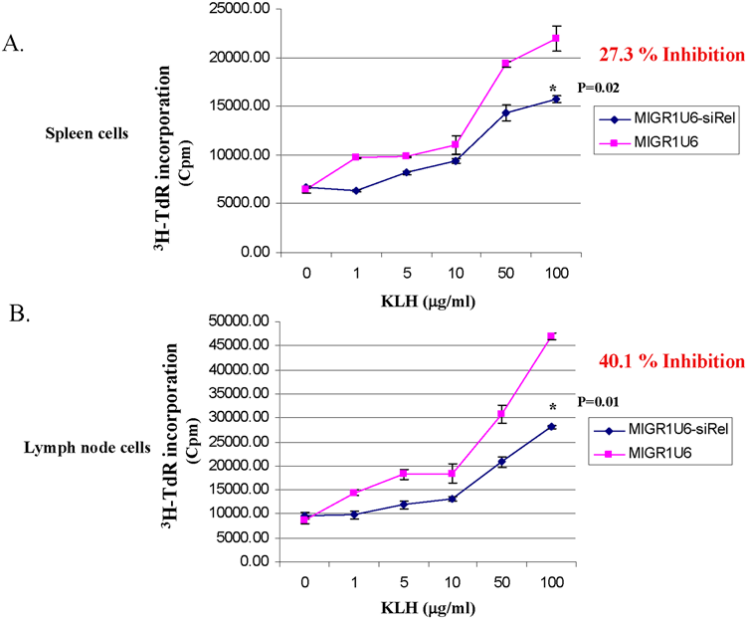
In vivo silencing c-Rel results in impaired proliferative response to antigenic stimulation. A group of 8–10 mice per experiment were immunized via hind footpad injection of KLH (100 µg) emulsified with CFA (Calbiochem, La Jolla, CA) at the ratio of 1∶1. After 9 days, total lymphocytes isolated from spleens (a) and draining lymph nodes (b) were cultured for 60 hr with various concentrations of KLH. Proliferation was measured by monitoring ^3^H-thymidine incorporation.

c-RelKO mice have impaired immune responses to a variety of antigens, including foreign protein antigens, auto-antigens, and allo-antigens [18], [19], [21], [22], [48]–[50], [93]–[95]. To validate that c-Rel-siRNA mimics the biological effect of the c-RelKO, we tested the bone marrow chimeric mice for proliferative responses to KLH as a foreign protein antigen. The chimeric mice transduced with either c-Rel-siRNA or control viruses were immunized with KLH (100 µg) through the hind footpad. Nine days later, lymphocytes were isolated from spleen and lymph node and cultured with KLH at increasing concentrations. Lymphocyte proliferation was monitored by thymidine incorporation 48 hours later. Our data showed that both splenic and lymph node lymphocytes derived from the c-Rel silencing group exhibited a significantly impaired T cell proliferative response to antigen re-stimulation as compared to that of the control group (**Fig. 5**). C-Rel silencing in splenocytes and lymph node cells led to a respective 27.3% and 40% inhibition in proliferative responses to 100 ug/ml KLH stimulation. Collectively, our data suggest that in vivo silencing of the c-Rel molecule can be achieved by delivering c-Rel-specific siRNA with retroviral mediated transduction and that partial c-Rel-knockdown in vivo results in a detectable reduction in lymphocyte proliferative responses to a protein antigen.

## Discussion

In this report, we demonstrate that retrovirus-mediated delivery of c-Rel specific siRNA results in significantly reduced cell survival and cell cycle progression for both B cell lymphoma and primary B cells. In vivo c-Rel knockdown further impairs an immune responses to antigenic stimulation, similar to the phenotypic response of the c-Rel knockout mice. Our data thus validate c-Rel as a target, and the use of c-Rel-siRNA as a potential intervention tool for the treatment of B cell tumors, inflammation, and autoimmune diseases.

Previous studies have shown that the Wehi-231 B cell tumor line expresses constitutively active NF-kB activity primarily composed of c-Rel and p50 [13], [76]–[86]. These cells are dependent on NF-kB for survival. Blocking the NF-kB activity leads to decreased expression of Bcl-X and c-myc, growth arrest, and apoptosis [77], [78], [80], [88]. Our studies using c-Rel-siRNA further validate the particularly important role of c-Rel in maintaining the survival of Wehi-231 cells, functioning presumably by blocking c-Rel-regulated anti-apoptotic molecules including Bcl-X, Bfl-1, Mcl-1, and c-myc [29], [60], [62], [64], [85], [88], [89], [96]–[100]. Our future plan is to test the c-Rel-siRNA on other B cell tumors with constitutive c-Rel activation, including diffuse large B cell lymphoma, multiple myeloma, and chronic lymphocytic leukemia, in order to expand the uses of c-Rel-siRNA as potential therapy for these tumors.

The inhibitory effect of c-Rel-siRNA on B lymphocyte proliferative responses as well as antigen-mediated immune responses essentially mimics the phenotype of the c-Rel knockout mice, thus validating the specific knockdown of c-Rel molecules by the siRNA. Our studies also suggest a practical application of the c-Rel-siRNA as a potential treatment for autoimmune diseases resulting from hyperactive immune responses to auto-antigens. The experiments presented in this report demonstrate that even reducing c-Rel expression in a sub-fraction of lymphoid and myeloid cells is sufficient to dampen T cell proliferative responses to the specific KLH antigen by 27–40%. These results suggest that blocking c-Rel even partially may be sufficient to subdue unwanted hyperactive immune responses, without compromising the host's defenses to microbial infection. In fact, the c-RelKO mouse studies have shown that, while these mice are protected from developing allergic inflammation, autoimmune diseases (EAE, Type I diabetes, collagen induced arthritis), their innate immune responses to pathogens (e.g. influenza virus, Toxoplasma gondii, Listeria monocytogenes) remains largely intact [17]–[22], [52]–[54]. These findings suggest that blocking c-Rel pharmacologically would not cause global immunosuppression, since innate immunity against pathogens remains intact in c-RelKO mice.

The involvement of IKK/NF-κB in a variety of human diseases has suggested that IKK/NF-κB is a potential therapeutic target. While IKKβ inhibitors could represent the first Rel/NF-kB-targeted therapy, systemic toxicity is a major concern for this class of inhibitors, because of the ubiquitous expression of IKK/NF-κB. Actually, early clinical trial data from an IKKβ inhibitor reported it to be rather toxic to cancer patients. This is likely attributed to the ubiquitous expression of p65 in all tissues as well p65's role in maintaining basal survival of many cell types in vivo. As suggested from early studies on the IKKβ and p65 knockout mice, both mice die at an early embryonic stage, due to hepatocyte cytotoxicity [26], [27], [101]. By comparison, c-Rel knockout mice are viable. Systemic suppression of c-Rel activity in mice protects against the development of autoimmune diseases, and shows no measurable adverse effects on development, metabolism, reproduction, or life-span. Since drug safety is an important feature for treating patients with chronic diseases, we propose an alternative strategy to tackle the Rel/NF-kB pathway without causing systemic toxicity: by targeting c-Rel.

The c-Rel-siRNA based inhibitors, if successfully developed, will offer several advantages over IKKβ inhibitors. (i) The c-Rel inhibitor will have significantly reduced toxicity compared to IKKβ inhibitors, as supported by knockout mouse studies. (ii) Blocking c-Rel suppresses the expression of multiple inflammatory cytokines and inhibits the growth of B cell tumors. (iii) The IKKβ inhibitor-based therapy will be ineffective toward tumors with genetic alterations downstream of IKK (e.g. IkB mutation, Rel TF overexpression). The c-Rel inhibitors, however, will circumvent such limitations.

Ultimately, our goal is to develop c-Rel-siRNA as a potential therapy for inflammatory disease, autoimmune disease, and B cell tumors. Although siRNA delivery remains a challenge, recent advances in this field have show significant improvement with regard to oligo stability and delivery systems [1], [2], [69]–[72]. For example, siRNA stability in serum can be improved significantly by incorporating 2′-O-methyl modification and phosphorothioate linkage into the siRNA backbone [1], [2]. Various formulation and delivery systems could be tested for delivery synthetic Rel-siRNA into tumors or target tissues by conjugation with cholesterol and peptides, or formulation with liposomes, lipoplexes, PEG, polyethylenimide (PEI), atelocollagen, or protamine-antibody [1], [2], [102]–[106]. Several clinical trials on small interfering RNA (siRNA)-based drugs are currently ongoing to target viral disease and macular degeneration [1], [2]. Thus, RNAi-mediated therapies hold promise for the treatment of diseases resulted from aberrant activation of a particular gene and its biological pathway.

## Materials and Methods

### Ethics Statement

The ethical use of animals in this study has been reviewed and approved by the Institutional Animal Care and Use Committee of the Weill Medical College of Cornell University (protocol #0304-112A, 0606-505A).

### Construction of siRNA expressing vectors

The method used for the construction of siRNA expression vector has been described (17). Briefly, Mouse U6 promoter was amplified by PCR from mouse genomic DNA using the following oligos: sense: 5′ GGAAGATCTATCCGACGCCGCCATCTCTA and antisense: 5′ GTGGAATTCGTTAAC GAAGACCACAAACAAGGCTTTTCTCCAA. Within the sense oligo sequence, *Bgl* II target sequence was underlined. Within the antisense oligo sequence, the first underlined sequence represents an *EcoR* I restriction site and the second is a *Bbs* I site. The PCR product was cloned into the *Bgl* II and *EcoR* I sites of pEGFP-C3 vector (Clontech) to generate a new vector, pEGFP-mU6-1 [73], [74].

The siRNA oilgos for c-Rel were designed as follows: upper strand: 5′ **TTTG**GTGTGAAGGGCGATCAGCAGGTTCAAGAGACCTGCTGATCGCCCTTCACACTTTTTC ; lower strand: 5′AATTGAAAAAGTGTGAAGGGCGATCAGCAGGTCTCTTGAACCTGCTGATCGCCCTTCACAC. Oligos were heated at 95°C for 5 minutes and then annealed at 37°C for one hour. Annealed sequence was ligated into the *Bbs* I and *EcoR* I sites of pEGFP-mU6-1 vector. Then, the siRNA expressing cassette was cut with *Bgl* II and *Hpa* I and subcloned into the MIGR1 vector to generate the MIGR1mU6-siRel vector [73], [74]. This vector will co-express siRNA transcript and green fluorescent protein (GFP).

### Preparation of retrovirus and determination of virus titer

Packaging of retrovirus was performed as described [75], [107]. Briefly, the MIGR1mU6-siRel plasmid or the MIGR1mU6 control plasmid was cotransfected with pHIT123 and P^CGP^ into 293T cell using calcium phosphate method. At 48 hours post transfection, the supernatant was harvested and assayed for viral titer by infection on NIH3T3 cells. The retrovirus supernatant was stored at −80°C for future use.

### In vitro silencing of c-Rel

To test the silencing effect of c-Rel siRNA expressing retrovirus, 2×10^5^ of NIH3T3 cells were seeded in 6-cm dishes. After culture for 24 hours, 100 µl of retrovirus (5×10^6^/ml) was added into 3T3 cells in the presence of polybrene (4 µg/ml). At 48 hours post-infection, cells were harvested and monitored by flow cytometry for the infection efficiency. The expression of c-Rel at protein level was tested by Western blot using c-Rel specific polyclonal antibody.

### In vitro infection of Wehi-231 cells

To test the effects of c-Rel silencing on Wehi-231 cell survival and cell cycle progression, 2 ml of the cells (2×10^6^/ml) were seeded in a 6-well plate and cultured with anti-CD40 (10 µg/ml) for 48 hours in the presence of polybrene (4 µg/ml) and different dosages of the c-Rel siRNA expressing retrovirus and the control virus (0.3125, 0.625, 1.25, and 2.5×10^6^). Cells were harvested and analyzed by PI staining [73], [74] for cell survival and cell cycle progression.

### In vitro infection of primary B cells

To test the effects of c-Rel silencing on B cell response, primary B cells were isolated from mouse spleen and were stimulated for 24 hr with anti-CD40 (10 µg/ml) before addition of the retroviruses. Cells were harvested at 48 hr post-infection and were monitored by propidium iodide (PI) staining analysis for cell survival and cell cycle progression, or by Ki-67 staining for cell proliferation.

### Generation of siRNA-expressing bone marrow chimeric mice

Generation of the chimeric mice was performed as described (17). Briefly, Donor mice (57BL/6, female, 8–10 weeks old) (The Jackson Labs, USA) were injected with 5-fluorouracil (5-FU, 250 mg/kg weight) per animal. Four days later, bone marrow cells (BMCs) were isolated from tibias and femurs of the mice and were cultured at a concentration of 2×10^6^/ml in 2 ml in 6-well plate with cytokine cocktail containing IL3 (6 ng/ml), IL6 (10 ng/ml) and SCF (100 ng/ml). After 24 hours, retrovirus supernatant was added into the BMCs and cultured for an additional 4–6 days. Cells were then collected and injected into lethally irradiated mice (850 Rad) through tail vein. Bone marrow chimeras were analyzed at 4–8 weeks post-bone marrow transfer (BMT). Cell reconstitution in each immune organ was monitored by flow cytometry for the percentage of GFP positive cells.

### Analysis of KLH-specific responses

For anti-KLH T cell responses, mice were immunized via hind footpad injection of KLH (100 µg) emulsified with CFA (Calbiochem, La Jolla, CA) at the ratio of 1∶1. After 9 days, the splenocytes and cells from draining lymph node were separately isolated and were cultured for 60 hr in various concentrations of KLH in RPMI 1640 medium supplemented with 10% FCS. Proliferation was measured by the addition of ^3^H-thymidine for the last 12 hours.

### Ki-67 staining

Ki-67 staining was performed as described [73], [74]. Briefly, 1×10^6^ cells harvested from the cell culture were washed twice with PBS and then fixed for 20 min in 0.5 ml of fixation buffer (eBioscience, San Diego, CA). After washing with PBS, cells were permeabilized at 4°C for 10 min in permeabilization buffer and then stained with PE-conjugated anti-Ki-67 antibody (PharMingen) for 30 min. Ki-67 expression cells were quantified by flow cytometry.
